# Predictors of clinical response to immunotherapy with or without radiotherapy

**DOI:** 10.1007/s13566-015-0219-2

**Published:** 2015-09-19

**Authors:** Susan M. Hiniker, Holden T. Maecker, Susan J. Knox

**Affiliations:** Department of Radiation Oncology, Stanford Cancer Center, Stanford University, 875 Blake Wilbur Drive, Stanford, CA 94305-5847 USA; Department of Microbiology and Immunology, Stanford University, Stanford, CA USA

**Keywords:** Radiation, Immunotherapy, Abscopal effect, Predictors of response, Biomarkers

## Abstract

Success with recent immunotherapies has resulted in previously unattainable response rates, as well as durable responses in diseases with historically poor prognoses. The combination of radiation therapy and immunotherapy has been a recent area of active investigation, with exciting results in a subset of patients. However, patient characteristics predictive of probable benefit from therapy and clinically meaningful biomarkers indicative of the early development of an antitumor immune response have yet to be identified. What is needed is a better way to predict which patients are likely to benefit from therapy, which would allow those patients unlikely to benefit from immunotherapy to be spared potentially futile therapies, thereby avoiding unnecessary risks of toxicity and costly treatment. Here, we summarize the early data on predictors of clinical response to immunotherapy, and to immunotherapy in combination with radiation.

## Introduction

Success with recent immunotherapies has resulted in previously unattainable response rates, as well as durable responses in diseases with historically poor prognoses. The combination of radiation therapy and immunotherapy has been a recent area of active investigation, with exciting results in a subset of patients. However, patient characteristics predictive of probable benefit from therapy and clinically meaningful biomarkers indicative of the early development of an antitumor immune response have yet to be identified. What is needed is a better way to predict which patients are likely to benefit from therapy, which would allow those patients unlikely to benefit from immunotherapy to be spared potentially futile therapies, thereby avoiding unnecessary risks of toxicity and costly treatment. Here, we summarize the early data on predictors of clinical response to immunotherapy, and to immunotherapy in combination with radiation.

## Recent advances in immunotherapy in treating malignancy

There has been significant recent interest in strategies designed to modulate the immune system in order to elicit and enhance an antitumor immune response. One of the early successes in this area has been in the targeting of cytotoxic T-lymphocyte-associated protein-4 (CTLA-4). CTLA-4 is a molecule expressed by activated T cells that competes with CD28 signaling on T cells, with its activation resulting in decreased T cell activation and proliferation. Ipilimumab, a monoclonal antibody directed against CTLA-4, was the first drug to show improved overall survival in patients with advanced melanoma [17] and has also shown single-agent activity in other malignancies. However, toxicity associated with ipilimumab is not insignificant and many patients do not respond to therapy. Preclinical and early clinical studies targeting the programmed death-1 receptor (PD-1), another T cell coinhibitory receptor, have shown even better response rates and lower toxicity than ipilimumab. PD-1 is expressed on activated T and B cells and has two identified ligands, programmed death-ligand 1 (PD-L1) and programmed death ligand-2 (PD-L2). Its primary ligand is PD-L1, expressed on a subset of hematopoietic and nonhematopoietic cells, which has been reported to be regulated by pro-inflammatory cytokines [39]. Multiple reports of anti-PD-1 therapy have shown promising results in the clinic in treating patients with advanced melanoma and other malignancies. In a study primarily composed of heavily pretreated patients with advanced melanoma and renal cell carcinoma, as well as patients with non-small cell lung cancer (NSCLC), castrate-resistant prostate cancer, and colorectal cancer (CRC), patients were treated with the anti-PD-1 targeted therapy nivolumab. Twenty-eight percent of patients experienced durable objective tumor responses. A subset analysis of patients with NSCLC revealed an objective response rate of 18 % [40].

Other studies have shown similarly promising results. For example, a large phase I trial with the anti-PD-1 antibody MK-3475 (pembrolizumab) had a response rate of 38 % in patients with advanced melanoma [13]. Another study of pembrolizumab showed an overall response rate of 26 % in patients who had experienced progression of disease on ipilimumab [33]. In the largest study of PD-1-directed therapy published to date, the KEYNOTE-006 randomized phase III trial, 834 patients with advanced melanoma received either pembrolizumab every 2 or 3 weeks or ipilimumab every 3 weeks. Both pembrolizumab groups had better PFS and OS as compared with the ipilimumab group, with less high-grade toxicity [34]. Finally, in a randomized double-blind study of 142 patients with BRAF V600E wild-type metastatic melanoma who had not previously received treatment, patients were randomized to ipilimumab plus or minus concurrent and adjuvant nivolumab. Patients who received combination therapy, compared to ipilimumab alone, had a better objective response rate to therapy (61 vs 11 %, *p* < 0.001), and better progression-free survival (not reached vs 4.4 months, *p* < 0.001), with an acceptable safety profile [28]. While these response rates are promising, it is clear that not all patients benefit from this immunotherapy. It is therefore important to be able to identify those patients likely to respond to treatment. It is hoped that results from ongoing trials will elucidate patient or tumor characteristics that are predictive of a high likelihood of response.

## Promising combinations of immunotherapy and radiation

Multiple groups have recently published case reports of abscopal responses in distal tumor sites (outside of the radiation therapy field) following local radiotherapy in combination with immunotherapy [11, 15, 26]. Reynders et al. reviewed the current data on the abscopal effect, consisting of one retrospective clinical study and a total of 23 case reports. In these reports, the median time to abscopal response was 5 months, with a median of 13 months after abscopal response before disease progression or end of follow-up [31]. In the single retrospective clinical study, 21 patients who progressed after ipilimumab alone were then treated with palliative radiotherapy. Median time to progression from the first dose of ipilimumab was 4 months (range 2.3–6 months), and median time from start of ipilimumab to radiation was 5 months (range 3.4–8 months). Of 21 patients, 11 (52 %) showed an abscopal effect including two patients with stable disease. In the patients who had an abscopal effect, median overall survival was increased versus those without an abscopal effect (22.4 vs 8.3 months, *p* = 0.002). Of note, these patients were treated with sequential, rather than concurrent therapy in this study, but it is likely that there may have been residual effects of the ipilimumab at the time of irradiation [12, 31]. In addition, in a recent phase I clinical trial performed at the University of Pennsylvania, 22 patients with metastatic melanoma were treated with hypofractionated radiation to a single lesion in combination with four cycles of ipilimumab. In terms of best clinical response, 18 % of patients had a partial response and 18 % had stable disease. Sixty-four percent of patients did not respond to this combination therapy and had progressive disease [42].

The mechanism responsible for the abscopal response is thought to be related to the effector T cell response, as animal models have shown that it is not possible to induce an abscopal response in athymic mice. Increased levels of IL-12, IFN-γ, and IL-2 also play an important role in the cytotoxic T cell response and associated abscopal effect [3]. A variety of preclinical and clinical studies have been performed to try to better elucidate the underlying mechanism of action of the abscopal response [16]. A number of preclinical studies have demonstrated that combination therapy with radiation and CTLA-4 blockade can lead to a significant survival benefit compared with monotherapy alone [5]. In patients, immunologic correlates of the abscopal effect were reported initially by Postow et al., in which they report clinical responses to radiation and concurrent ipilimumab correlating with an increase in antibody titers to NY-ESO-1 and other tumor-associated antigens, as well as increase in CD4+ T cell and myeloid lineage activation, and corresponding decline in myeloid-derived suppressor cells [27]. These results have led to an ongoing phase II randomized trial of patients (with at least two separate measurable sites of disease), in which patients are randomized to ipilimumab alone or with radiation, with immune monitoring of T cell and B cell responses to melanoma-associated tumor antigens [6].

## Predictors of response to therapy

While there is considerable excitement surrounding initial response rates and durable responses to immunotherapy, as well as combination therapy with immunotherapy and radiation therapy, at present it is not known how to identify those patients who are likely to respond to treatment. There are also no validated biomarkers to detect the development of a potentially clinically meaningful antitumor immune response in treated patients. This is currently an active area of investigation. Early reports indicated that patients with metastatic melanoma who have less than 1000 lymphocytes per cubic millimeters, particularly after the first or second course of ipilimumab, have a worse prognosis [21]. The most consistently reported predictor of response to PD-1 pathway blockade is pretreatment tumor PD-L1 expression. Taube et al. found that infiltrating immune cells were geographically associated with PD-L1 expression and that expression of PD-L1 on tumor cells and immune infiltrates represents an “immune-active tumor milieu.” Tumor PD-L1 expression correlated with response to anti-PD-1 therapy [39].

Tumeh et al. reported that in patients treated with pembrolizumab for advanced melanoma, responding patients had proliferation of intratumoral CD8+ T cells, which directly correlated with radiographic reduction in tumor size. Responding patients had higher pretreatment numbers of CD8, PD1, and PD-L1 expressing cells, both at the tumor margin and inside of the tumors [41]. A predictive model was created based on CD8 expression at the invasive margin and was validated in an independent set of 15 patients, with authors concluding that tumor regression after PD-1 blockade requires preexisting CD8+ T cells, which are negatively regulated by a PD-1/PD-L1-mediated adaptive resistance [41]. In this study, the preexisting density of CD8+ T cells was more closely correlated with response to anti-PD-1 therapy than was PD-L1 expression. Also of note, responders had significantly higher levels of pSTAT1+ at the invasive margin in the area of CD8+ T cell infiltration before and during treatment, with significantly higher levels of pSTAT1 expression during treatment compared to baseline [41].

In an effort to better determine which patients with metastatic melanoma will respond to CTLA-4 blockade and may benefit from treatment with ipilimumab, Snyder et al. performed whole-exome sequencing from tumors and matched blood samples to analyze somatic mutations and potential neoantigens generated from these mutations. They found that a high mutational load was associated with benefit from treatment with CTLA-4 blockade, and also found a strong neoantigen landscape in patients who respond to CTLA-4 blockade, with some homology to viral and bacterial antigens. This neoantigen landscape was validated in another set of 39 patients. Response peaked at 60 weeks following treatment [37]. Similarly, in a study of patients with NSCLC, higher numbers of mutations and a corresponding increase in neoantigens was found to correlate with clinical response in patients treated with anti-PD-1 pembrolizumab [32].

In a recent report by Johnson et al., outcomes of 229 patients with melanoma treated with immune therapies were compared by tumor genotype. They found that benefit from anti-PD-1/PD-L1 was significantly greater in patients with NRAS mutations. NRAS-mutant melanoma was found to have higher PD-L1 expression compared with other genotypes, though this was not statistically significant [19].

 Data presented at the SITC 2014 meeting included phase IIb results from the TIME study examining TG4010 immunotherapy combined with initial chemotherapy in advanced NSCLC. TG4010 is a modified vaccine vector encoding the entire tumor antigen MUC1 and IL-2 [18, 30]. Previous phase I data had shown a survival advantage with the addition of TG4010 to chemotherapy in patients with normal levels of circulating activated NK cells (CD16+ CD56+ CD69+), but not in patients with high levels of NK cells. A prospective study incorporating the previous results enrolled patients with advanced MUC1-expressing NSCLC to receive TG4010 versus placebo with first-line platinum-based chemotherapy. In a subgroup analysis of 221 patients, patients with normal levels of pretreatment-activated NK cells and patients with nonsquamous histology showed a significant improvement in PFS, with a trend toward OS benefit. Patients with both normal levels of pretreatment-activated NK cells and nonsquamous histology benefitted the most. A large phase III trial is now ongoing to further investigate this finding [18, 30].

Another study examining biomarkers and characteristics of response to PD-1 immune checkpoint blockade in NSCLC patients was also recently reported at the 2014 SITC meeting. Only two markers of response have thus far been shown to correlate with higher response rates to PD-1 blockade in NSCLC patients: PD-L1 expression and smoking status, with smokers more likely to respond. However, these are not specific markers, and PD-L1-negative patients and never smokers may sometimes respond to PD-1 blockade. In addition, PD-L1 expression may be inconsistent between the primary site and metastatic sites of disease [1]. A recent study showed that in the context of combination immunotherapy for melanoma, PD-L1 expression in the tumor and absolute lymphocyte count did not appear to be predictive markers of tumor response [28]. The authors hypothesize that in combination therapy, it is possible that anti-CTLA-4 therapy drives T cell infiltration into tumors, creating a more optimal environment for anti-PD-1 therapy to work [18].

The benefits of anti-PD-1/anti-PD-L1 therapy have been reported in other diseases in addition to melanoma and NSCLC. For example, treatment with the anti-PD-L1 antibody MPDL3280A has been shown to reduce metastatic lesions in patients with renal cell carcinoma and urothelial bladder cancer [14]. Herbst et al. reported that responses were seen in patients with tumors of multiple types including melanoma, renal cell carcinoma, and NSCLC expressing high levels of PD-L1, particularly when PD-L1 was expressed by tumor-infiltrating immune cells. They note that response to therapy was associated with T-helper type 1 gene expression, CTLA4 expression, and the absence of CX3CL1 in baseline tumor specimens [14]. In gene expression studies, baseline IFN-γ and CXCL9 were significantly higher in study patients who had a partial or complete response (PR/CR) to treatment versus those with progressive disease, and IFN-γ, IDO1, and CXCL9 were significantly higher in study patients with melanoma who had a PR/CR to treatment versus those with progressive disease. Patients who progressed through treatment most commonly lacked PD-L1 upregulation by tumor cells or tumor-infiltrating immune cells [14]. In examining biomarkers found in the blood, they found a rise in ITAC and IL-18, as well as an increase in activated cytotoxic T lymphocytes and decrease in IL-6. However, these changes did not clearly distinguish responders from nonresponders.

Powles et al. also examined the safety and activity of the anti-PD-L1 antibody MPDL3280A and found a high response rate in patients with metastatic bladder cancer, with durable ongoing responses in 16 of 17 responders. They found no cytokines to be predictive of response. They did observe transient elevations in IL-18 and IFN-γ by cycle 2, as well as an increase in proliferating CD8+ cells in treated patients, but these changes were not specific to the responders [29]. High levels of PD-L1 in the tumor were again found to predict better response to therapy.

Recent data presented at the ASCO 2015 meeting also supports the use of immunotherapy in a variety of tumor types and suggests potential biomarkers of response. In the CheckMate 057 phase III randomized trial, patients with advanced nonsquamous NSCLC who had progressed on platinum-based doublet chemotherapy were randomized to receive nivolumab or docetaxel. The trial was closed early due to a survival advantage in the nivolumab group, with a median overall survival (OS) of 12.2 versus 9.4 months in the docetaxel group. All subgroups of patients had a survival benefit from treatment, except for those with tumors with EGFR mutations. PD-L1 was a strong predictor of response to treatment with nivolumab, with median OS of 17.2, 18.2, and 19.4 months for patients whose tumors had at least 1, 5, and 10 % of cells staining positive for PD-L1, respectively [25].

Other results presented at ASCO support a potential role for PD-1 blockade in advanced hepatocellular carcinoma (HCC). In a phase I/II study of nivolumab in advanced HCC, 19 % of 42 patients had a response with tumor reduction greater than 30 %, with two complete responses, and durable responses lasting beyond 12 months for 50 % of patients. Treatment was well tolerated, including among patients with hepatitis B or C infections. Overall survival at 1 year was 62 % [9].

Results from an expansion cohort of KEYNOTE-012 trial also presented at ASCO showed a benefit to pembrolizumab in patients with recurrent or metastatic squamous cell carcinoma of the head and neck. The overall objective response rate was 24.8 %, and 57 % of patients experienced some tumor shrinkage, with roughly equivalent response rates in HPV-positive and HPV-negative tumors. Responses were often long-lasting, and less than 10 % of patients experienced significant side effects from treatment [35].

Results from a phase II study presented at ASCO showed that tumors with mismatch repair (MMR) deficiencies respond better to treatment with pembrolizumab than those that were MMR-proficient. Among three cohorts of patients, including 25 patients with MMR-deficient CRC, MMR-proficient CRC, and MMR-deficient cancers including uterus, stomach, prostate, duodenum, and bile ducts, those patients with MMR-deficient tumors had a response rate to pembrolizumab of approximately 60 %, as compared with 0 % for patients with MMR-proficient tumors. Of note, MMR deficiency is associated with high mutational burden, consistent with previous studies showing better response to anti-PD-1 therapy in tumors with a high number of mutations [9].

## Response to combination therapy

In other settings, biomarkers have been used to predict response to systemic therapy plus radiation. Elevated neutrophil/lymphocyte ratio before chemoradiation has been reported to predict poor pathologic tumor response and survival after preoperative chemoradiation for rectal cancer [20]. Preclinical markers of response to therapy support p53 as playing an important role in the abscopal response following local tumor irradiation [2, 38]. In the Postow et al. report on markers of abscopal responses, the presence of NY-ESO-1 antibodies after radiotherapy enhanced the efficiency of ipilimumab. Following radiotherapy, the antibody titer rose by 30-fold [28]. Also noted was an increase in CD4+ T cells after radiation therapy, as well as a decrease in myeloid-derived suppressor cells prior to identification of an abscopal effect. Following radiation and ipilimumab, titers against melanoma antigen-A2 also increased [27]. These findings led to an ongoing phase II trial of patients with at least two separate measurable sites of disease, with patients randomized to ipilimumab alone or with radiation, with immune monitoring of T cell and B cell response to melanoma-associated tumor antigens [6].

Blockade of TGF-β has been shown to contribute to the induction of abscopal effects, and also to overcome local immunosuppression [8]. In a preclinical model, Demaria et al. found a synergistic benefit to TGF-β blockade combined with local tumor irradiation, with increase in tumor infiltrating lymphocytes. Loss of therapeutic benefit was seen after depletion of CD4 or CD8 T cells. Upregulation of immune system pathway genes occurred in response to combination therapy, and the addition of anti-PD-1 therapy provided further benefit [8]. Importantly, expression of coinhibitory molecules such as PD-L1 have been shown to be induced in tumor cells after local high-dose radiation therapy [7], which has potential implications for the optimal sequencing of these therapeutic agents.

In studies of patients with metastatic hepatocellular carcinoma, there have been reports of changes in serum cytokines before and after radiation therapy. Ohba et al. reported an abscopal effect occurring with decreased AFP and concomitant increase of TNF-α after radiation therapy [24]. Nakanishi et al. also studied serum cytokines before and after radiation therapy, and did not find an increase in TNF-α, but did note an increase in IL-18 before and after radiotherapy [23].

In a phase I clinical trial of 22 patients with metastatic melanoma at the University of Pennsylvania, patients were treated with ipilimumab plus local radiation therapy to a single lesion, and the unirradiated lesion/s were assessed for response. The best response to therapy was a partial response in 18 % of patients and stable disease in another 18 % of patients. Twyman-Saint Victor et al. then used a corresponding melanoma mouse model and found that low CD8+/Treg ratio predicted resistance of melanoma to anti-CTLA4 therapy and radiation, and profiling of these tumors revealed high levels of tumor PD-L1. The defect in CD8+ T cell accumulation in resistant tumors was related to increased PD-L1 expression on the melanoma cells. Genetic silencing of PD-L1 caused the melanomas to again become sensitive to anti-CTLA-4 therapy and radiation. Addition of anti-PD-L1 IgG increased the number of active CD8+ T cells [42]. The authors reported that combining PD1, PD-L1, and CTLA4-mediated immune checkpoint inhibition with radiation promoted effective antitumor immunity through a distinct, nonredundant mechanism. As noted by Twyman-Saint Victor et al. and reviewed by Leavy, complete responses were CD8+ T cell dependent, and failure to increase the ratio of CD8+ CD44+ T cells to Tregs (CD8/Treg ratio) within the tumor infiltrating lymphocyte population predicted resistance [22, 42]. They found that the lack of increase in the CD8+/Treg ratio in resistant tumor cells was due to a failure of CD8+ T cells to accumulate in tumors, since Tregs did decrease, as in sensitive tumors, and high PD-L1 expression on tumor cells was found to cause reduced CD8+ T cell accumulation. Markers of exhaustion and activation in peripheral T cells, as well as changes in the CD8+/Treg ratio as above in the peripheral blood, were shown to be predictive of response in mice to this immunotherapy. In the clinical trial patients, high PD-L1 expression on pretreatment tumor biopsies was associated with persistent T cell exhaustion and disease progression after combination treatment, suggesting that combination therapy targeting both PD-L1 and CTLA-4 may be important for the optimization of this immunotherapy approach [22, 42].

## Current studies

Animal studies are ongoing to characterize the cellular immune responses to systemic therapies that may allow for identification of candidate genes and cellular toxicity pathways that are important for response [10]. For example, Frick et al. isolated splenocytes from 36 isogenic strains of mice in the development of a drug screening platform and examined interstrain differences in the viability of immune cells following chemotherapy, with phenotypes quantified with flow cytometry. As expected, more targeted agents BEZ-235 and selumetinib were less toxic to normal immune cells than standard chemotherapeutic anthracycline agents. They also found that heritability of the viability of immune cells after exposure to therapy was higher for anthracyclines than targeted agents between generations [10].

There are multiple ongoing clinical trials testing the combination of immunotherapy and radiation, as recently summarized by Crittenden et al. [4]. A variety of studies are also examining ways to convert nonresponders to responders following immunotherapy [26]. An immunotherapy biomarkers task force has been established to further investigate biomarkers of response in order to better select patients for therapy, and ultimately learn how to steer the immune system in the direction of meaningful and durable clinical responses [36].

## Summary

**Fig. 1:**
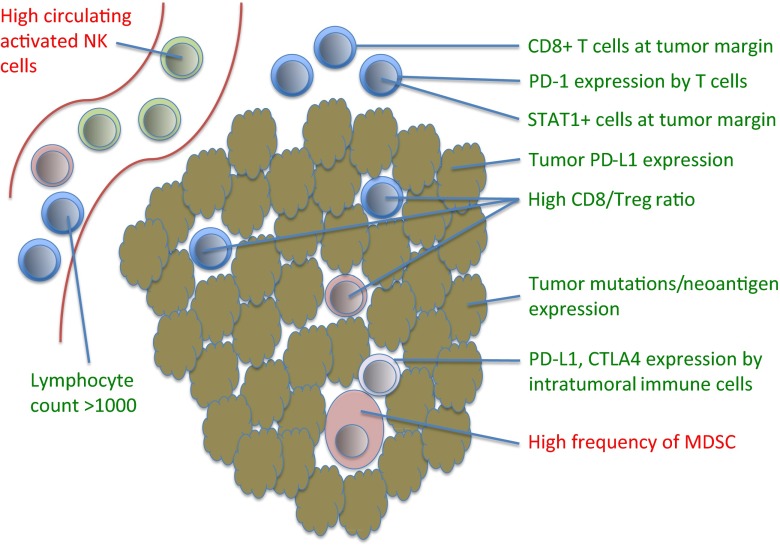
Potential positive (*green*) and negative (*red*) predictors of outcome in anti-PD-1 immunotherapy. Some biomarkers have also been shown for other types of immunotherapy, though there is variation by immunotherapy and tumor type. Most of these biomarkers are measured from immunohistochemistry (IHC) of tumor biopsy sections; activated NK cells and lymphocyte counts are derived from peripheral blood

In summary, the early data on biomarkers of response to immunotherapy suggest that pretreatment tumor PD-L1 levels, as well as preexisting numbers of CD 8+ T cells and levels of pSTAT1+ cells at the tumor margin may be important predictors of response (Fig. 1). However, the PD-L1 level may be less important in the context of combination therapy with anti-CTLA-4 and anti-PD-1 therapy. Higher tumor mutational load and MMR deficiency also appears to correlate with response to therapy. Other potential markers of response with more limited data at this time include IFN-γ, IDO1, CXCL9, and IL-18. The CD8+/Treg ratio appears to play a role in response to treatment and may be related to PD-L1 levels. Further work is necessary to more fully elucidate biomarkers of response in order to better predict which patients are likely to respond to immunotherapies, and to assess early patient response to treatment.
